# Associations of lipid levels susceptibility loci with coronary artery disease in Chinese population

**DOI:** 10.1186/s12944-015-0079-1

**Published:** 2015-07-25

**Authors:** Xue-bin Wang, Ya-di Han, Ning-hua Cui, Jia-jia Gao, Jie Yang, Zhu-liang Huang, Qiang Zhu, Fang Zheng

**Affiliations:** Center for Gene Diagnosis, Zhongnan Hospital of Wuhan University, Wuhan, 430071 Hubei China; Department of Clinical Laboratory, Children’s Hospital of Zhengzhou, Zhengzhou, 450053 Henan China; Department of Gasteroenterology, Provincial Hospital Affiliated to Shandong University, Jinan, 250021 Shandong China

**Keywords:** Coronary artery disease, *HNF1A* rs1169288, *MADD-FOLH1* rs7395662, Gensini scores

## Abstract

**Background:**

Recent genome-wide association studies (GWAS) have identified several single nucleotide polymorphisms (SNPs) that were associated with blood lipid levels in Caucasians. This study investigated whether these loci influenced lipid levels and whether they were associated with the risk of coronary artery disease (CAD) and its angiographic severity in Chinese population.

**Methods:**

Six SNPs were genotyped in 1100 CAD cases and 1069 controls using the high-resolution melting (HRM) method. Coronary atherosclerosis severity was assessed by the vessel scores and the Gensini scoring system.

**Results:**

Among the 6 SNPs and the genetic risks scores (GRS), the minor alleles of *HNF1A* rs1169288 (odd ratio (OR) = 1.18, 95 % confidence interval (CI) 1.05–1.33, P = 0.006) and *MADD-FOLH1* rs7395662 (OR = 1.20, 95 % CI 1.07–1.36, P = 0.002) as well as the GRS (P = 1.06 × 10^-5^) were significantly associated with increased risk of CAD after false discovery rate (FDR) correction. The vessel (P = 0.013) and Gensini scores (β = 0.113, P = 0.002) differed among CAD patients with different SNP rs1169288 C > T genotypes. The multiple linear regression analyses using an additive model revealed that the minor allele C of SNP rs1169288 (β = 0.060, P = 0.001) and the GRS (β = 0.033, P = 3.59 × 10^-4^) were significantly associated with increased total cholesterol (TC) levels, the minor allele A of SNP rs7395662 (β = -0.024, P = 0.007) and the GRS (β = -0.013, P = 0.004) were significantly associated with decreased high-density lipoprotein cholesterol (HDL-c) levels.

**Conclusions:**

The present study demonstrated that SNPs rs1169288, rs7395662 and the GRS were significantly associated with lipid levels and the risk of CAD in Chinese population. Furthermore, the allele C of SNP rs1169288 increased the odds of coronary atherosclerosis severity.

**Electronic supplementary material:**

The online version of this article (doi:10.1186/s12944-015-0079-1) contains supplementary material, which is available to authorized users.

## Background

Coronary artery disease (CAD), one of the most common cardiovascular disease [1], is associated with high morbidity and mortality and remains one of the most common causes of death globally [2]. A main underlying pathology of CAD is atherosclerosis, a process of cumulative deposition of lipoproteins in the arteries supplying blood to the heart that eventually leads to impaired or absent blood supply and myocardial infarction (MI) [3]. Atherosclerosis has numerous genetic and environmental risk factors [4], and abnormalities of plasma lipids and lipoproteins are heritable risk factors for CAD, with heritability estimates ranging from 40–60 % for total cholesterol (TC), triglyceride (TG), low-density lipoprotein cholesterol (LDL-c), high-density lipoprotein cholesterol (HDL-c) and 30–60 % for CAD [5]. Recently, independent genome-wide association studies (GWAS) have identified several loci that influence blood lipid levels and CAD risk in Caucasians [6–11]. However, the associations between these novel single nucleotide polymorphisms (SNPs), lipid levels and the risk of CAD were not well established in Chinese population. Otherwise, few studies have examined the utility of genetic risk scores (GRS) to identify Chinese subjects at increased CAD risk [12, 13].

The locus rs7395662 on chromosome 11p11.2, was first identified as a strongly lipid-associated locus according to a GWAS in Caucasians [6]. This study also identified SNP rs12670798 as a novel lipid-associated locus located in intron of the dynein axonemal heavy chain 11 (*DNAH11*) gene [6]. SNP rs9411489, which is located 4.3 kb downstream of the ABO blood group (*ABO*) gene, was also found to be associated with lipid levels in a GWAS of Europeans [10]. Then a large-scale association analysis in individuals of European descent identified the following three variants associated with lipid levels and CAD risk: SNPs rs1169288, rs1495741 and rs10128711, which located in or near the hepatocyte nuclear factor 1-α (*HNF1A*) gene, N-acetyltransferase 2 (*NAT2*) gene and SPT2, Suppressor of Ty, domain containing 1 (*SPTY2D1*) gene, respectively [11].

In this study, we aimed to examine the associations of these 6 lipid-associated variants (individually and in combination) with lipid levels and CAD risk in Chinese population. Additionally, a cross-sectional study on the associations between these six SNPs and the severity of coronary atherosclerosis has been conducted.

## Materials and methods

### Study population

Study participants were recruited from Zhongnan hospital of Wuhan University and Asia Heart Hospital between January 2011 and October 2014. The present case–control study involved 1100 CAD cases and 1069 non-CAD controls. CAD cases were diagnosed based on ≥ 50 % luminal stenosis in at least one major coronary arteries or their major branches by standard coronary angiography. Non-CAD controls were the subjects without detectable stenosis by coronary angiography and healthy population controls without diagnosis of CAD, hypertension and diabetes mellitus (DM) by routine physical examinations. Otherwise, to assess the effects of these six SNPs on lipid levels in our control group, subjects who were undergoing lipid-lowering medication or dyslipidemia were also excluded from the controls.

Fasting concentrations of the plasma glucose, C-reactive protein (CRP), TC, TG, LDL-c and HDL-c were measured using standard methods [14]. Other clinical data collected from study participants included age, sex, history of smoking, alcohol intake, body mass index (BMI), weight status (overweight and obesity status), hypertension, diabetes mellitus, dyslipidemia and pharmacological therapy (including lipid/glucose/blood pressure lowering drug treatment) status. BMI was defined as weight divided by height in square meters (kg/ m^2^). Overweight and obesity were defined as BMI ≥ 25 and ≥ 30, respectively [15]. Hypertension was diagnosed based on usage of ongoing therapy for hypertension, systolic blood pressure (SBP) of ≥ 140 mmHg or diastolic blood pressure (DBP) of ≥ 90 mmHg [16]. DM was defined as ongoing therapy for diabetes or fasting plasma glucose (FPG) levels of ≥ 7.0 mmol/L, or with plasma glucose levels of ≥ 11.1 mmol/L [17]. This study was approved by ethnics committee of Zhongnan hospital of Wuhan University and met the declaration of Helsinki.

### Scoring of coronary angiogram

Coronary angiograms were scored according to the vessel and Gensini scores. The vessel scores were defined as the number of vessels having ≥ 50 % stenosis. In the Gensini scoring system [18], the narrowing of the coronary artery lumen is scored 1 for 0–25 % stenosis, 2 for 26–50 %, 4 for 51–75 %, 8 for 76–90 %, 16 for 91–99 % and 32 for 100 %. Each stenosed segment was then weighted from 0.5 to 5, depending on the functional significance of the area supplied by that segment. These scores were multiplied by the coefficient defined for each coronary artery and segment, and the results were then summed.

The scores were independently assessed by two experienced interventional cardiologists who were blinded to the procedural data and clinical outcomes. The κ for inter-observer variability that was used to estimate the vessel and Gensini scores were 0.98, 0.88, respectively, whereas the κ for intra-observer variability that were 0.99, 0.93, respectively. Any disagreements regarding the scores were resolved by consensus.

### SNP selection and genotyping

We first looked for previously reported SNPs associated with lipid levels from GWAS with replication evidence in Caucasian populations [6, 7, 10, 11]. Then, SNPs that have been associated with CAD risk in Chinese (such as *SORT1* rs599839 [19], *CILP2* rs16996148 [19], *DOCK7*rs737337 [20], etc.) were excluded. Finally, to derive adequate power (>80 %), only SNPs with minor allele frequencies (MAF) >15 % in Chinese individuals in the HapMap database were selected, including *HNF1A* rs1169288, *MADD-FOLH1* rs7395662, *NAT2* rs1495741, *DNAH11* rs12670798, *ABO* rs9411489, *SPTY2D1* rs10128711 (Table 1).

**Table 1:** Characteristics of 6 SNPs in our study

SNP	Chr	Position^a^	Nearby gene	Risk allele	RAF		HWE (P value)	
					Case	Control	Case	Control
rs1169288	12q24.31	120978847	*HNF1A*	C	0.472	0.430	0.178	0.132
rs7395662	11p11.2	48497341	*MADD-FOLH1*	A	0.527	0.481	0.410	0.115
rs1495741	8p22	18415371	*NAT2*	G	0.591	0.557	0.174	0.928
rs12670798	7p15.3	21567734	*DNAH11*	C	0.495	0.485	0.238	0.208
rs9411489	9q34.3	133279427	*ABO*	T	0.213	0.219	0.208	0.321
rs10128711	11p15.1	18611437	*SPTY2D1*	C	0.473	0.463	0.385	0.063

*Chr* chromosome, *RAF* risk allele frequency, *HWE* Hardy-Weinberg equilibrium

^a^Information for chromosome position is based on NCBI genome build 38.2

**Fig. 1:**
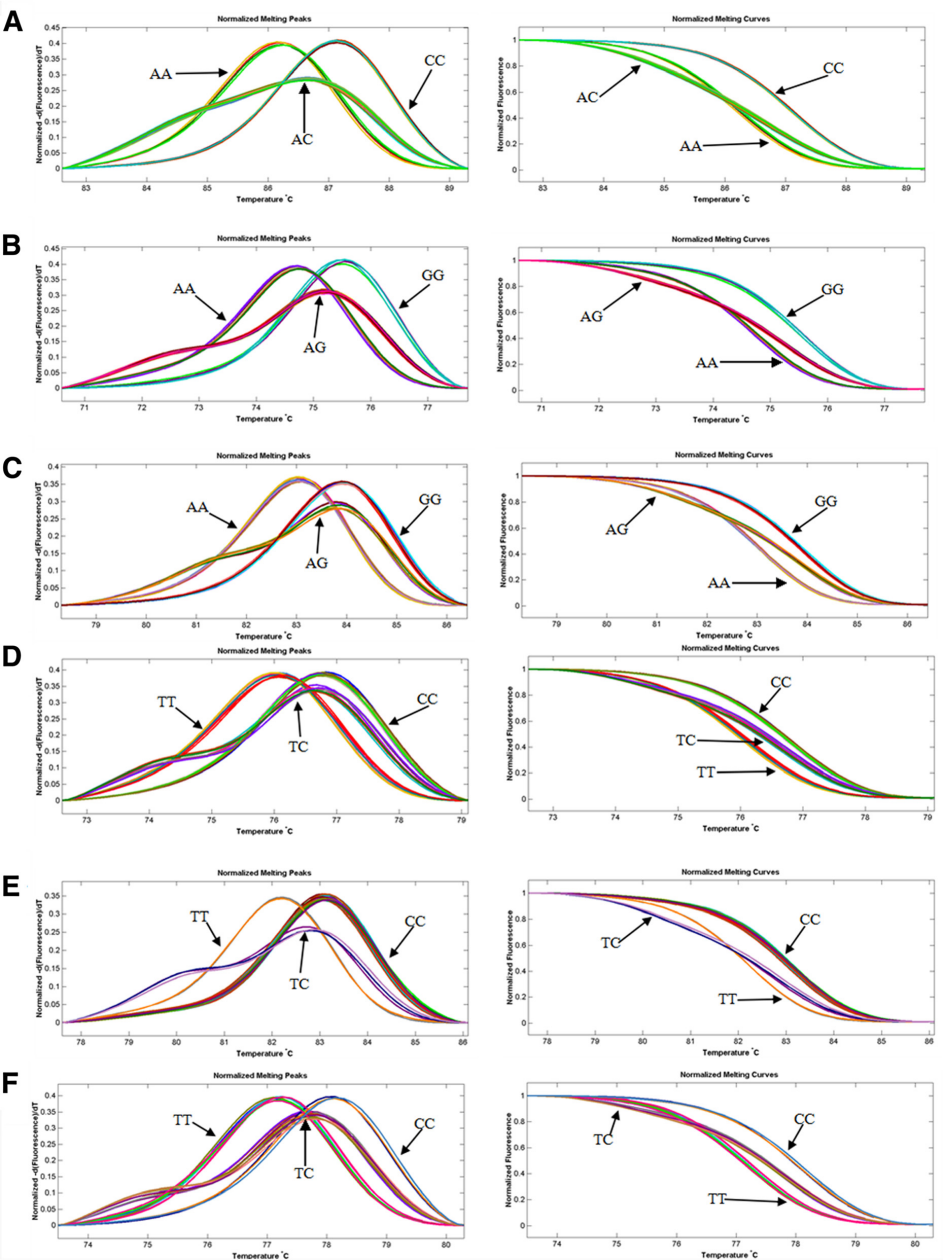
HRM plots for different genotypes of six SNPs. The normalized melting peaks are given in the left column, and the normalized melting curves are given in the right column. Arrows indicate the genotypes. The representative HRM plots of *HNF1A* rs1169288, *MADD-FOLH1* rs7395662, *NAT2* rs1495741, *DNAH11* rs12670798, *ABO* rs9411489, *SPTY2D1* rs10128711 are shown in **a**, **b**, **c**, **d**, **e** and **f**, respectively

Genomic DNA was isolated from peripheral blood white cells using the phenol/chloroform method. SNPs were genotyped using a LightScanner 96 High Resolution Melt (HRM) system (Idaho Technology, Salt Lake City, UT, USA). PCR reaction for genotyping was performed in a total of 10 μL PCR volume containing 1 μL of LC green dye, 5 pmol of each primer, 25 ng of genomic DNA, 2 μL of 10 × PCR buffer with 1.5 mmol/L MgCl_2_, 2 mmol of deoxynucleotide triphosphates, and 1 unit of Taq polymerase. HRM analysis is employed post-PCR to thermally denature the small amplicons and measure the subtle differences in melting temperature (Tm) between different genotypes [21]. Wild-type and homozygous mutant samples are distinguished by Tm shifts. Heterozygous samples are best distinguished from homozygous, not by Tm, but by altered curve shape (Fig. 1). Genotyping call rates for all 6 SNPs were > 99 %. Primer details and product lengths were shown in Additional file 1. For each SNP, a total of 24 cases and controls were randomly selected to be sequenced, and the genotypes were confirmed. DNA sequence analysis was performed with forward and/or reverse primers using the BigDye Terminatior v3.1 Cycle Sequencing Kits on an ABI PRISM 3100 genetic Analyzer (Applied Biosystems, Foster City, CA, USA).

### Statistical analyses

Continuous variables with normal distribution were expressed as mean ± SD, and differences between groups were compared by the Student’s t-tests. Variables with skewed distributions were ln-transformed before analyses. Categorical variables were represented as percentages and were tested by the *χ*^2^ tests, which were also used to test for deviation of genotype distributions from Hardy-Weinberg equilibrium (HWE) .

In the case–control analyses, the allele frequencies in cases and controls were compared using the *χ*^2^ tests. The genotypic associations of 6 SNPs with CAD risk were assessed by the logistic regression analyses under different models of inheritance (additive, recessive and dominant) after adjusting for age, sex, smoking, alcohol intake, weight status, hypertension, type 2 diabetes, dyslipidemia and pharmacological therapy covariates. The weighted GRS of CAD were the weighted sum across three significant (uncorrected) SNPs (rs1169288, rs7395662 and rs1495741) combining the odds ratios (ORs) and doses of risk alleles based on an additive model [22, 23]. The effects of SNPs on plasma lipid levels were assessed by the multiple linear regression analyses under an additive model. The weighted GRS of lipid levels were also assessed as the sum of doses of the risk alleles weighted by the β coefficients at the polymorphisms [24, 25]. For CAD cross-sectional study, the vessel scores were compared among the genotypes of six SNPs using the linear-by-linear association *χ*^2^ test and the *χ*^2^ test. Otherwise, the associations between the Gensini scores and six SNPs were assessed by treating the Gensini scores as quantitative traits (the multiple linear regression) and using median case–control methods (the logistic regression) [26, 27]. Using the false discovery rate (FDR) method [28], multiple testing correction for the associations with lipid levels, CAD risk and coronary atherosclerosis severity were conducted separately. The P _FDR_ value was calculated by multiplying its P value by the number of tests performed and then divided by the rank order of each P value (where rank order 1 is assigned to the smallest P value). An FDR of 0.05 was used as a critical value to assess whether P_FDR_ value was significant.

Statistical analyses were conducted by SPSS 17.0 (SPSS, Inc., Chicago, Illinois, USA). Power analysis was carried out using Power and Sample Size Program 3.0 (Vanderbilt University, Nashville, TN, USA).

## Results

### Characteristics of the study population

Clinical characteristics of the study population are listed in Table 2. Age and gender distributed similarly between the two groups. Blood pressure (BP), plasma concentrations of glucose, TC, TG, CRP levels, BMI and the prevalence of overweight and obesity were significantly higher, while HDL-c levels were significantly lower in the case group than those of the control group. There were no significant differences for LDL-c levels and the rate of smoking and alcohol intake between the two groups. The genotypes of 6 SNPs were all in HWE (P > 0.05, Table 1).

**Table 2:** Clinical characteristics of participants in the case–control study

Characteristics	CAD patients (*n* = 1100)	Controls (*n* = 1069)	P
Age, years	59.9 ± 8.7	59.4 ± 9.3	0.176
Male, n (%)	666 (60.5)	659 (61.6)	0.599
Current smoking, n (%)	415 (37.7)	373 (34.9)	0.170
Alcohol intake, n (%)	409 (37.2)	387 (36.2)	0.636
BMI, kg/m^2^	24.0 ± 3.23	23.1 ± 3.35	<0.001
Overweight, n (%)	341 (31.0)	278 (26.0)	0.010
Obesity, n (%)	183 (16.6)	99 (9.3)	<0.001
Hypertension, n (%)	647 (58.8)	0 (0)	<0.001
DM, n (%)	357 (32.5)	0 (0)	<0.001
Hyperlipidemia, n (%)	234 (21.3)	0 (0)	<0.001
Antihypertensive treatment, n (%)	601 (54.6)	0 (0)	<0.001
Glucose-lowering treatment, n (%)	323 (29.4)	0 (0)	<0.001
Lipid-lowering treatment, n (%)	226 (20.5)	0 (0)	<0.001
SBP, mmHG	128.1 (126.9–129.3)	121.6 (120.9–122.2)	<0.001
DBP, mmHG	83.5 (82.6–84.4)	80.7 (80.0–81.4)	<0.001
FPG, mmol/L	6.04 (5.94–6.15)	4.97 (4.94–4.99)	<0.001
TC, mmol/L	4.32 ± 1.03	4.03 ± 0.42	<0.001
TG, mmol/L	1.53 ± 1.01	1.05 ± 0.36	<0.001
LDL-c, mmol/L	2.78 ± 1.04	2.74 ± 0.26	0.256
HDL-c, mmol/L	1.22 ± 0.20	1.27 ± 0.22	<0.001
CRP, mmol/L	4.54 ± 7.45	1.15 ± 1.78	<0.001
Gensini scores	22.3 (21.2, 23.5)		

Data are expressed as mean ± SD or geometric mean (95 % confidence interval)

*CAD* coronary artery disease, *BMI* body mass index, *DM* diabetes mellitus, *SBP* systolic blood pressure, *DBP* diastolic blood pressure, *FPG* fasting plasma glucose, *TC* total cholesterol, *TG* triglyceride, *LDL-c* low-density lipoprotein cholesterol, *HDL-c* high-density lipoprotein cholesterol, *CRP* C-reactive protein

### Associations with lipid levels

We investigated the associations between six novel SNPs and lipid levels in 1069 healthy control subjects (Table 3). Under an additive model adjusted for age, sex, alcohol intake, smoking and weight status, the minor allele C of *HNF1A* rs1169288 was significantly associated with increased TC levels (β = 0.060, SE = 0.018, P = 0.001) and decreased HDL-c levels (β = -0.018, SE = 0.009, P = 0.046); The minor allele A of *MADD-FOLH1* rs7395662 was associated with decreased HDL-c levels (β = -0.024, SE = 0.009, P = 0.007). In addition, the lipid levels GRS based on an additive model showed positive associations with increased TC levels (β = 0.033, SE = 0.009, P = 3.59 × 10^-4^) and decreased HDL-c levels (β = -0.013, SE = 0.005, P = 0.004). After FDR correction for multiple testing, the statistical associations for SNP rs1169288 with TC levels (P_FDR_ = 0.014), SNP rs7395662 with HDL-c levels (P_FDR_ = 0.049) and the GRS with TC (P_FDR_ = 0.010) and HDL-c levels (P_FDR_ = 0.037) remained significant.

**Table 3:** Associations of 6 SNPs with lipid levels in 1069 healthy control subjects

SNP	Chr	Locus	Risk/non-risk allele	TG (mmol/L)			TC (mmol/L)			LDL-c (mmol/L)			HDL-c (mmol/L)			TC			TG			LDL-c			HDL-c		
				β (SE)	P	P_FDR_^a^	β (SE)	P	P_FDR_^a^	β (SE)	P	P_FDR_^a^	β (SE)	P	P_FDR_^a^	β (SE)	P	P_FDR_	β (SE)	P	P_FDR_	β (SE)	P	P_FDR_	β (SE)	P	P_FDR_
rs1169288	12	*HNF1A*	C/A	0.013 (0.016)	0.388	0.747	*0.060 (0.018)*	*0.001*	*0.014*	0.027 (0.015)	0.074	0.345	-0.018 (0.009)	0.046	0.258	*0.060 (0.018)*	*0.001*	*0.014*	0.009 (0.015)	0.551	0.812	0.026 (0.015)	0.084	0.344	-0.019 (0.009)	0.036	0.202
rs7395662	11	*MADD-FOLH1*	A/G	-0.015 (0.015)	0.317	0.683	-0.020 (0.018)	0.256	0.639	-0.002 (0.015)	0.874	0.979	*-0.024 (0.009)*	*0.007*	*0.049*	-0.020 (0.018)	0.268	0.670	-0.015 (0.015)	0.323	0.674	-0.007 (0.015)	0.643	0.857	*-0.024 (0.009)*	*0.007*	*0.049*
rs1495741	8	*NAT2*	G/A	0.003 (0.016)	0.867	0.979	0.015 (0.018)	0.400	0.746	0.022 (0.015)	0.141	0.448	0.007 (0.009)	0.514	0.795	0.015 (0.018)	0.842	0.953	0.003 (0.016)	0.858	0.953	0.022 (0.015)	0.149	0.464	0.006 (0.009)	0.510	0.804
rs12670798	7	*DNAH11*	C/T	0.009 (0.015)	0.568	0.795	0.021 (0.018)	0.243	0.639	-0.004 (0.015)	0.800	0.974	-0.006 (0.009)	0.426	0.746	0.019 (0.018)	0.287	0.670	0.008 (0.015)	0.581	0.813	-0.004 (0.015)	0.795	0.953	-0.008 (0.009)	0.387	0.677
rs9411489	9	*ABO*	T/C	-0.013 (0.038)	0.733	0.933	0.025 (0.044)	0.568	0.795	0.001 (0.036)	0.998	0.998	0.002 (0.022)	0.910	0.980	0.019 (0.022)	0.373	0.677	-0.018 (0.019)	0.337	0.674	0.017 (0.018)	0.953	0.953	0.001 (0.011)	0.928	0.953
rs10128711	11	*SPTY2D1*	C/T	0.017 (0.015)	0.274	0.639	-0.006 (0.018)	0.725	0.933	0.023 (0.014)	0.111	0.444	0.001 (0.009)	0.957	0.992	-0.007 (0.018)	0.692	0.881	0.017 (0.015)	0.274	0.667	0.025 (0.015)	0.086	0.344	0.001 (0.009)	0.933	0.953
GRS				0.005 (0.008)	0.518	0.795	*0.033 (0.009)*	*3.59 × 10* ^*-4*^	*0.010*	0.009 (0.006)	0.144	0.448	*-0.013 (0.005)*	*0.004*	*0.037*	*0.033 (0.009)*	*2.76 × 10* ^*-4*^	*0.008*	0.005 (0.008)	0.517	0.804	0.009 (0.006)	0.146	0.464	*-0.014 (0.005)*	*0.003*	*0.028*

Italic values are statistically significant after FDR correction

*Chr* chromosome, *TC* total cholesterol, *TG* triglyceride, *LDL-c* low-density lipoprotein cholesterol, *HDL-c* high-density lipoprotein cholesterol; β (SE), effect size (standard error)

^a^P-value from Benjamini-Hochberg method control for false discovery rate (FDR)

### Associations with the risk of CAD

Among the 6 SNPs, there were significant differences in the allele frequencies between CAD cases and controls for *HNF1A* rs1169288 (OR = 1.18, 95 % CI = 1.05–1.33, P = 0.006), *MADD-FOLH1* rs7395662 (OR = 1.20, 95 % CI = 1.07–1.36, P = 0.002) and *NAT2* rs1495741 (OR = 1.15, 95 % CI = 1.02–1.30, P = 0.024) (Table 4). After correction for multiple testing, the minor alleles of SNPs rs1169288 (P_FDR_ = 0.030) and rs7395662 (P_FDR_ = 0.025) were still significantly associated with increased risk of CAD. Based on a MAF of 43.0 % in our control group and type I error of 0.05, the recruited samples could provide 84.1 % power to detect genetic effect with an allelic OR of 1.20 for SNP rs1169288. For SNP rs7395662, our population had a power of 84.5 % to detect the association with CAD risk assuming an allelic OR of 1.20 and a MAF of 48.1 % in our control group.

**Table 4:** Allelic and genotypic associations of 6 SNPs with CAD risk in our case–control study

SNP	Genotype	Frequency (n)		Allelic comparison			Additive model			Dominant model			Recessive model		
		Cases	Controls	OR (95 % CI)	P	P_FDR_^a^	OR (95 % CI)	P	P_FDR_^a^	OR (95 % CI)	P	P_FDR_^a^	OR (95 % CI)	P	P_FDR_^a^
rs1169288	AA	318	359	*1.18 (1.05, 1.33)*	*0.006*	*0.030*	*1.25 (1.06, 1.45)*	*0.008*	*0.033*	*1.45 (1.13, 1.86)*	*0.004*	*0.025*	1.22 (0.93, 1.61)	0.149	0.310
C vs A	AC	526	500												
	CC	256	210												
rs7395662	GG	239	301	*1.20 (1.07, 1.36)*	*0.002*	*0.025*	*1.22 (1.05, 1.43)*	*0.012*	*0.043*	*1.50 (1.15, 1.97)*	*0.003*	*0.025*	1.18 (0.91, 1.52)	0.212	0.379
A vs G	AG	562	508												
	AA	299	260												
rs1495741	AA	195	209	1.15 (1.02, 1.30)	0.024	0.075	1.16 (0.99, 1.36)	0.058	0.145	1.09 (0.82, 1.45)	0.546	0.696	1.31 (1.03, 1.66)	0.029	0.081
G vs A	AG	510	529												
	GG	395	331												
rs12670798	TT	259	293	1.08 (0.96, 1.22)	0.188	0.362	1.07 (0.92, 1.25)	0.398	0.622	1.19 (0.97, 1.47)	0.098	0.223	1.13 (0.87, 1.46)	0.354	0.590
T vs C	TC	568	512												
	CC	270	261												
rs9411489	CC	688	657	0.96 (0.83, 1.11)	0.621	0.739	0.98 (0.84, 1.15)	0.821	0.892	1.00 (0.83, 1.20)	0.998	0.998	0.97 (0.64, 1.49)	0.904	0.942
T vs C	TC	355	355												
	TT	57	57												
rs10128711	TT	313	321	1.04 (0.92, 1.17)	0.557	0.696	1.05 (0.92, 1.21)	0.446	0.656	1.09 (0.88, 1.36)	0.483	0.671	1.05 (0.85, 1.31)	0.659	0.749
C vs T	TC	534	499												
	CC	253	244												

Italic values are statistically significant after FDR correction

*OR* (95 % CI), odds ratio (95 % confidence interval)

^a^P-value from Benjamini-Hochberg method control for false discovery rate (FDR)

To explore the potential inheritance patterns, three models of inheritance including additive, dominant and recessive models were explored for each SNP (Table 4). Results from the logistic regression analyses indicated that SNPs rs1169288 and rs7395662 were significantly associated with the risk of CAD under both additive (OR = 1.25, 95 % CI = 1.06–1.45, P = 0.008, P_FDR_ = 0.033 for SNP rs1169288; OR = 1.22, 95 % CI = 1.05–1.43, P = 0.012, P_FDR_ = 0.043 for SNP rs7395662) and dominant models (OR = 1.45, 95 % CI = 1.13–1.86, P = 0.004, P_FDR_ = 0.025 for SNP rs1169288; OR = 1.50, 95 % CI = 1.15–1.97, P = 0.003, P_FDR_ = 0.025 for SNP rs7395662) after adjusting for age, sex, current smoking, alcohol intake, weight status, hypertension, type 2 diabetes, dyslipidemia and pharmacological therapy covariates. Significant genotypic association was identified between SNP rs1495741 and CAD risk under a recessive model (OR = 1.31, 95 % CI = 1.03–1.66, P = 0.029), but it was not sufficient robust to withstand the FDR correction (P_FDR_ = 0.081). Associations between other three SNPs and CAD risk were not significant in neither allelic nor genotypic association analyses.

**Fig. 2:**
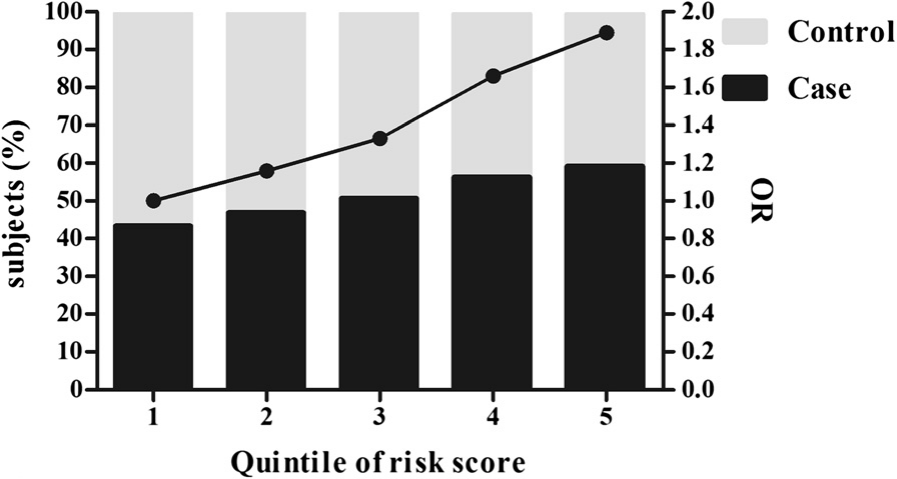
GRS categories and risk for CAD. Black and gray bars represent the subjects of case and control in each quintile, respectively. Solid dots depict the respective odds ratios for CAD risk when different quantiles compared with quintile 1

To examine the cumulative effect of three associated SNPs (rs1169288, rs7395662 and rs1495741) on the risk of CAD, the weighted GRS was calculated. The mean GRS of CAD cases was significantly higher than that of controls (P = 1.06 × 10^-5^, P_FDR_ = 2.65 × 10^-4^). Moreover, the logistic regression analyses revealed that subjects with the top quintile GRS were associated with 1.89-fold increased risk of CAD compared with those having the low quintile GRS (after adjusting for 9 covariates, Fig. 2 and Additional file 2).

## Angiographic severity of coronary atherosclerosis

The associations between six SNPs and the angiographic severity in CAD cases were evaluated by investigating the vessel and Gensini scores. According to the number of significantly affected vessels, we observed a dose-dependent effect of genotypes of SNP rs1169288 on the vessel scores (*χ*^2^ test for linear-by-linear association: P = 0.013; *χ*^2^ test: P = 0.002, P_FDR_ = 0.012, Table 5).

**Table 5:** Associations of 6 SNPs with the vessel scores in the 1100 CAD patients

SNP	Diseased vessel scores (%)			*χ* ^2^ test			Linear-by- linear *χ* ^2^ test	
	1	2	3	*χ* ^2^ value	P	P_FDR_^a^	*χ* ^2^ value	P
rs1169288				*16.507*	*0.002*	*0.012*	*6.120*	*0.013*
AA	126 (33.9)	95 (32.3)	97 (22.4)					
AC	161 (43.3)	131 (44.6)	234 (53.9)					
CC	85 (22.8)	68 (23.1)	103 (23.7)					
rs7395662				5.620	0.229	0.458	0.888	0.346
GG	81 (21.8)	68 (23.1)	90 (20.7)					
AG	190 (51.1)	160 (54.4)	212 (48.9)					
AA	101 (27.1)	66 (22.5)	132 (30.4)					
rs1495741				3.071	0.546	0.655	1.580	0.209
AA	64 (17.2)	53 (18.0)	78 (18.0)					
AG	165 (44.4)	132 (44.9)	213 (49.1)					
GG	143 (38.4)	109 (37.1)	143 (32.9)					
rs12670798				8.320	0.081	0.243	0.005	0.944
TT	93 (25.1)	77 (26.3)	89 (20.6)					
TC	179 (48.2)	142 (48.5)	247 (57.0)					
CC	99 (26.7)	74 (25.2)	97 (22.4)					
rs9411489				0.337	0.987	0.987	0.106	0.745
CC	230 (61.8)	186 (63.3)	272 (62.7)					
TC	121 (32.5)	94 (31.9)	140 (32.3)					
TT	21 (5.7)	14 (4.8)	22 (5.0)					
rs10128711				3.126	0.537	0.655	2.750	0.097
TT	97 (26.0)	86 (29.2)	130 (30.0)					
TC	181 (48.7)	139 (47.3)	214 (49.3)					
CC	94 (25.3)	69 (23.5)	90 (20.7)					

Italic values are statistically significant after FDR correction

^a^P-value from Benjamini-Hochberg method control for false discovery rate (FDR)

For SNP rs1169288, the geometric mean (95 % CI) of the Gensini scores were 19.4 (17.6–21.3) in the genotype AA carriers, 23.3 (21.6–25.1) in the genotype AC carriers and 23.6 (21.2–26.3) in the genotype CC carriers, respectively. When we analyzed the Gensini scores as quantitative traits (Table 6), the minor allele C of SNP rs1169288 was significantly associated with higher Gensini scores after adjusting for 9 covariates (β = 0.113, SE = 0.036, P = 0.002, P_FDR_ = 0.012). When the allele frequencies of each SNP in the higher and lower Gensini scores of patients were compared in a case–control design (Table 6), significant association was obtained again between SNP rs1169288 and the Gensini scores (OR = 1.30, 95 % CI = 1.09–1.53, P = 0.003, P_FDR_ = 0.018). Otherwise, there were no significant associations between other five SNPs and the severity of CAD as assessed both by the vessel and Gensini scores.

**Table 6:** Associations of 6 SNPs with the ln-transformed Gensini socre in the 1100 CAD patients under additive model

SNP (risk allele)	Quantitative trait association			Median case–control association^a^				
				Risk allele frequency (%)				
	β (SE)	P^b^	P_FDR_^c^	High scores	Low scores	OR (95 % CI)	P^b^	P_FDR_^c^
rs1169288 (C)	*0.113 (0.037)*	*0.002*	*0.012*	*50.0*	*44.4*	*1.30 (1.09, 1.53)*	*0.003*	*0.018*
rs7395662 (A)	0.059 (0.038)	0.121	0.363	51.8	53.1	0.98 (0.82, 1.16)	0.768	0.768
rs1495741 (G)	0.006 (0.037)	0.872	0.872	60.2	58.0	1.10 (0.93, 1.31)	0.252	0.504
rs12670798 (T)	0.025 (0.038)	0.513	0.770	51.2	49.8	1.06 (0.89, 1.26)	0.500	0.750
rs9411489 (T)	0.017 (0.045)	0.709	0.851	22.0	20.7	1.05 (0.86, 1.29)	0.636	0.763
rs10128711 (C)	-0.036 (0.037)	0.329	0.658	46.0	48.5	0.89 (0.75, 1.05)	0.158	0.474

Italic values are statistically significant after FDR correction

*β (SE)* effect size (standard error), *OR* (95 % CI) odds ratio (95 % confidence interval)

^a^CAD patients were classified into two groups according to their Gensini scores using the median as a cutoff point: >20.5 for the high scores group and ≤20.5 for the low scores group. ^b^P values were obtained using the multivariate linear and logistic regression analyses with age, sex, smoking, alcohol intake, weight status, hypertension, diabetes and dyslipidemia and pharmacological therapy status as covariates. ^c^P-value from Benjamini-Hochberg method control for false discovery rate (FDR)

## Discussion

In this study, we have demonstrated that two SNPs (*HNF1A* rs1169288, *MADD-FOLH1* rs7395662) and the GRS were associated with lipid levels in Chinese population. The present study also indicated that these two SNPs and the GRS were associated with CAD risk after correction for multiple testing, and this is the first investigation demonstrating that SNP rs1169288 was significantly associated with the angiographic severity of coronary atherosclerosis.

SNP rs7395662 on chromosome 11p11.2 showed reliable evidence for associations with HDL-c levels and the risk of CAD in our study, which was consistent with the results from previous GWAS in Caucasians [6]. However, another association study including 727 Guangxi Han population demonstrated that this locus was significantly associated with TC, TG, HDL-c and LDL-c levels [29]. To our knowledge, the frequencies of G allele in the Guangxi Han subjects were significant lower than those in our Hubei control subjects (43.7 % vs 51.9 %) and also much lower than those in the HapMap CHB database (43.7 % vs 56.5 %), suggesting that the Chinese population may not be genetically similar. Apart from genetic background, such as sample size and different statistical method, may also contribute to the discrepancies among our study and other studies in Chinese population. The role that this SNP might play in lipoprotein metabolism is still unclear now. SNP rs7395662 is assigned as *MADD-FOLH1* locus of chromosome 11p, and the two genes flanking the locus, MAP-kinase activating death domain (*MADD)* gene and folate hydrolase 1 (*FOLH1*) gene have not been implicated in lipid metabolism. However, the liver X receptors alpha variant 1 (*LXRA*) gene, an orphan member of the nuclear receptor that regulate pathways central to lipid homeostasis [30, 31], is located just 0.5 kb telomeric of *MADD* gene. Moreover, polymorphisms of this gene have been associated with lipid levels and CAD risk in Caucasians [32]. So whether SNP rs7395662 is a functional one, or just a tagging one, needs to be determined by further functional studies.

Another SNP, rs1169288, which is a non-synonymous SNP in the coding region of *HNF1A* gene, has been reported to be associated with TC, LDL-c levels and CAD risk in a GWAS of Caucasians [11]. Reiner et al. also found significant associations of the minor allele C with higher LDL-c levels and increased risk of CAD in the two case–control samples from African -Americans and Caucasians [33]. In our study, the minor allele C of SNP rs1169288 was associated with higher TC levels and increased risk of CAD, but not associated with LDL-c levels in Chinese population. One possible reason of these differences is that our sample size is not enough to detect the association because of the subtle effect size of the individual variant on LDL-c levels. Another explanation is that different linkage disequilibrium patterns existed in different populations. After all, a number of studies have suggested that there may be genetic differences between the determinants of lipid profiles in different ethnicities [34, 35]. *HNF1A* gene, encoded the transcription factor hepatocyte nuclear factor 1-α, which regulated the transcription of numerous genes involved in lipid transport and metabolism [36]. SNP rs1169288 is located within the *HNF1A* dimerization domain that has been associated with decreased *in vitro* transcriptional activity of downstream target gene promoters [37]. Therefore, this variant may influence multiple atherosclerosis-related genes or their plasma products through effects on HNF-1α structure or function [33].

The Gensini scoring system is a well-used method for measuring the severity of coronary atherosclerosis, which is the primary pathophysiological process underlying CAD [38]. Because our 1100 Gensini scores were in accordance with normal distribution after ln-transformation, we performed quantitative trait association and median case–control association analyses for the six SNPs. For both analyses, the associations between the Gensini scores and SNP rs1169288 were significant even after FDR correction. Moreover, we also observed a dose-dependent effect of genotypes of this polymorphism on the vessel scores. Although the exact biological mechanism of these associations remained to be explored, our study provided evidence that SNP rs1169288 may contribute to the etiology of the severity of coronary atherosclerosis and played an important role in the atherosclerotic process.

To combine the relatively small effects of individual genes and to better consider the actual effect of each SNP on the trait [22], we calculated the weighted GRS based on the three significant (uncorrected) SNPs. The results indicated that each additional risk allele was associated with increased TC levels and decreased HDL-c levels even after multiple testing. More importantly, for weighted GRS of CAD, quintile 5 had a 1.89 times increased odds of CAD as compared to quintile 1, suggesting a potential predictive effect on CAD risk.

Limitations of our study merit consideration. First, the retrospective design of this study has inherent drawbacks and precludes causal inferences [39]. Second, the sample size of the present study may not enough either to detect a marginal effect from very low penetrance SNPs or to identify significant associations of the effect in different genetic model analyses. Third, the GRS were based on SNPs that were significantly associated with CAD risk in our study, and this process may resulted in the possible exaggeration of risk prediction [22]. Because the effect sizes of SNPs were calculated and tested in the same cohort. Finally, due to lack of clinical data, we have not examined the effects of SNPs and the GRS on more biomarkers such as lipoprotein (a), apolipoprotein A1 and apolipoprotein B, and on imaging measures, such as the extent of coronary atherosclerosis (the Sullivan Extent scores) and carotid intima-media thickness (CIMT).

## Conclusions

In summary, in a case–control study with 1100 CAD patients and 1069 controls, we have identified that two SNPs (*HNF1A* rs1169288, *MADD-FOLH1* rs7395662) as well as the GRS were significantly associated with TC, HDL-c levels and CAD risk, and correlation may exist between the *HNF1A* rs1169288 and severity of coronary atherosclerosis. Functional studies are required to explore the mechanisms governing these effects.

## Additional files

Additional file 1:
**Amplification primers utilized in the genotyping.** (XLS 19 kb) Additional file 2:
**Cumulative effects of associated variants on the risk of CAD.** (XLS 18 kb)
